# Severe Neurocysticercosis in an Immunocompetent Male Without Travel to an Endemic Region: A Case Report

**DOI:** 10.7759/cureus.34870

**Published:** 2023-02-11

**Authors:** Anuradha Sakhuja, Sharada KC, Joshua Wortsman, Dhan B Shrestha, Barun B Aryal, Vishal Kwatra, Larissa Verda

**Affiliations:** 1 Department of Internal Medicine, Mount Sinai Hospital, Chicago, USA

**Keywords:** cerebral cysticercosis, encephalitic neurocysticercosis, teniasis, seizure, neurocysticercosis

## Abstract

Neurocysticercosis is a neglected parasitic cause of seizures in the United States. It can have a wide array of presentations depending on the location and number of cysticercoids. The severity of symptoms varies with the location of the lesion in the brain and to the extent of the number of neurocysticercoids and host immune response. In the severe form of neurocysticercosis, it can present as an acute encephalitic picture. We present a case of severe neurocysticercosis in a patient without any significant travel history. Neurocysticercosis in nonendemic areas can be diagnostically challenging, given the lack of travel history as in our patient. Neurocysticercosis should be kept as a differential in all cases of seizures without prior history of seizure episodes.

## Introduction

Seizures have a variety of causes, including metabolic, neurologic, infectious, and drug-induced, and hence provide a diagnostic dilemma in evaluation during the first episode [1]. The infectious agents can be bacterial, viral, or parasitic, leading to seizures. Among the infectious causes of seizure, neurocysticercosis is one of the most common parasitic infections of the central nervous system in the endemic region. In areas where pig farming is practiced, especially in low-income nations, neurocysticercosis is endemic [2]. Although not endemic in the United States, neurocysticercosis accounts for 2.1% of all emergency department visits for seizures [3]. In the United States, it is particularly seen in immigrants and those with a travel history [4]. Neurocysticercosis has been identified as a neglected parasitic infection in the United States [3].

Here, we present a case report of encephalitic neurocysticercosis in a person native to Chicago without any travel history to the neurocysticercosis endemic region.

## Case presentation

A 40-year-old Hispanic male with no reported past medical history was brought in by ambulance after two episodes of witnessed seizures, each lasting for approximately 10 seconds. On presentation to the emergency department, the patient was found to be hypoxic, with oxygen saturation of 60% on room air, which increased to 96% on 15 L of O_2_ via a nonrebreather mask. He was unresponsive to painful stimuli with agonal respirations and tachycardia. Cough and gag reflexes were absent. He had bleeding from the oral cavity with injury to his tongue. He did not have any prior episodes of seizures and was not under any medications. The patient had lived all his life in Chicago and had no history of travel to regions endemic to neurocysticercosis.

The patient was intubated for airway protection and started on midazolam seizure dosing and fentanyl for sedation. His COVID-19 rapid test was positive. His labs showed leukocytosis and an acidotic state consistent with seizures. His urine drug screen was positive for cocaine metabolites. On admission, a computed tomography (CT) of the head demonstrated multiple areas of vasogenic edema throughout the supratentorial brain bilaterally, greater in the left anterior frontal lobe with multiple lesions. He was given a loading dose of levetiracetam and started on a maintenance dose thereafter.

**Figure 1:**
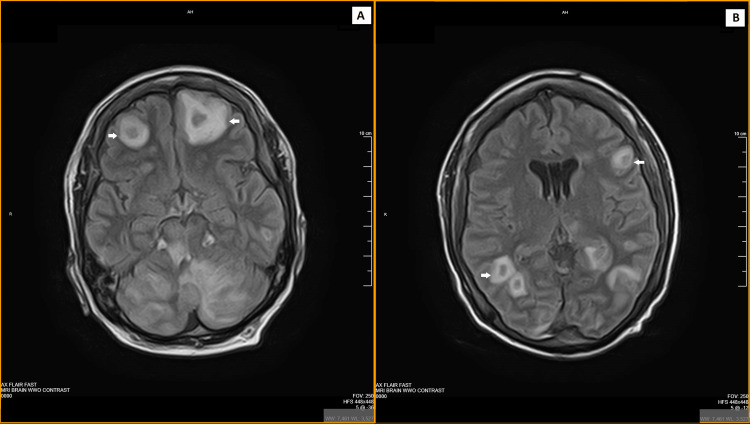
MRI brain on presentation with axial sections (flair) (both A and B) showing multiple ring-enhancing lesions (white arrows). MRI, magnetic resonance imaging

Given the CT head findings, a pan CT scan was obtained that did not show any significant abnormal findings. MRI brain showed innumerable ring-enhancing lesions throughout the supratentorial and infratentorial brain and in the midbrain and pons with the mass effect, particularly in the posterior fossa near the fourth ventricle and quadrigeminal plate cistern (Figure 1). Multidisciplinary teams, including Infectious disease, neurology, neurosurgery, and oncology services, were consulted. The patient was started on high-intensity steroids and levetiracetam. Serological labs, including *Treponema pallidum*, *Blastomyces*, *Cryptococcus*, cytomegalovirus (CMV), hepatitis panel, *Toxoplasma*, and tuberculosis, were sent, all of which came back negative. Then a lumbar puncture was performed, which did not show any notable findings. An echocardiogram was negative for any cardiac/valvular pathology.

Given the negative workup and high clinical imaging suspicion of neurocysticercosis, the decision was made to start empiric treatment with albendazole and praziquantel along with high-intensity steroids.

**Figure 2:**
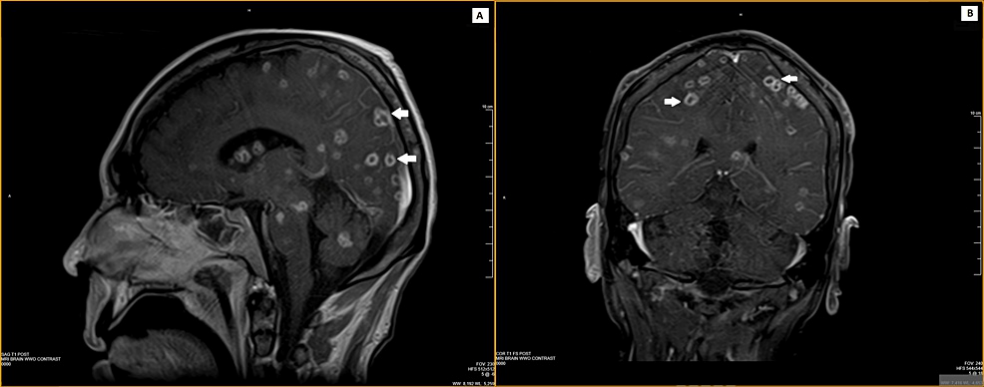
MRI brain before biopsy (T1-weighted) showing multiple ring-enhancing lesions (white arrows) on the (A) sagittal section and (B) coronal section. MRI, magnetic resonance imaging

When all serology came back negative for any significant findings, a decision was made to perform a brain biopsy. MRI brain was repeated before the brain biopsy, which showed innumerable small ring-enhancing lesions seen throughout the cerebral and cerebellar hemispheres, as well as the brainstem and cervical-medullary junction (Figure 2). Lesions increased in number from the previous imaging. He underwent a brain biopsy where larvae were seen and a cyst with larvae was removed intraoperatively. Ophthalmology evaluation was negative for intraophthlamic infection.

The patient underwent a tracheostomy during the hospital course due to ventilator-dependent respiratory failure. The hospital course was complicated by persistent thrombocytopenia not responding to a large amount of platelet transfusion. Eventually, he had improvement in mentation and was weaned off the ventilator. He continued to improve clinically, and after a month of hospital stay, he was discharged to a subacute rehab center. Levetiracetam was continued at discharge. He was subsequently discharged from the acute rehabilitation facility to the care of his family with the continuation of physical therapy. Subsequent brain imaging shows gradual resolution of lesions. During a follow-up visit one year after the initial presentation, he showed neurological improvement but reported persistent numbness of bilateral upper and lower extremities and right-hand tremors.

## Discussion

Cysticercosis is caused due to tissue infection from *Taenia solium*. Neurocysticercosis is an infection of the central nervous system by the larval stage of the parasite [5]. The manifestations of neurocysticercosis depend on the location of implantation of cysticercoids, which can be intraparenchymal or extra parenchymal [6]. Intraparenchymal neurocysticercosis typically presents with seizure, whereas extra-parenchymal neurocysticercosis presents with hydrocephalus and raised intracranial pressure [5].

Our patient had multiple intraparenchymal cysticercoids, leading to diffused cerebral edema, and presented with multiple seizures and a severe presentation. Neurocysticercosis can present with a wide variety of neurological presentations mimicking a wide range of diseases, especially in endemic regions [7]. In a retrospective study of an academic hospital in the United States, headaches and seizures were found to be the most common presenting complaint of neurocysticercosis [4].

The diagnosis of neurocysticercosis depends on the travel history or origin of the patient, endemicity of neurocysticercosis in the region, imaging findings, and serological tests [8,9]. Imaging studies can detect the viable phase, degenerative phase, and dead or calcified phase of parenchymal neurocysticercosis as well as the cysts of extra-parenchymal neurocysticercosis [10]. CT can better delineate the dead or calcified forms of intraparenchymal neurocysticercosis than MRI. However, for all other intra- and extra-parenchymal forms, MRI is superior to CT for the diagnosis of neurocysticercosis [11]. In our patient, CT could not identify the lesions, whereas MRI did show ring-enhancing lesions in multiple locations, which were not diagnosed with neurocysticercosis by the imaging. Furthermore, there was severe cerebral edema mimicking the acute encephalitic picture. The encephalitic form of neurocysticercosis occurs when the body reacts to a high load of *T. solium* [12]. Cysticercotic encephalitis is more common in children and females [13]. However, our patient is an adult male with the clinical picture.

Serological tests available for diagnosis of neurocysticercosis include enzyme-linked immunoelectrotransfer blot (EITB) and enzyme-linked immunosorbent assay (ELISA). EITB has better sensitivity than ELISA and is preferred for diagnosis [14]. However, EITB is not available readily. Because of a lack of travel history and being unable to diagnose from the imaging findings, our patient went for a brain biopsy where larvae were noted intraoperatively.

Albendazole along with praziquantel is recommended for the management of more than two viable cysts. Furthermore, corticosteroids should be given for the resolution of associated inflammation and cerebral edema [9]. Our patient improved both clinically and radiologically under the treatment and was successfully weaned off the ventilator. He underwent physical therapy with some improvement in neurological function. Subsequent imaging showed gradual resolution of the lesions. However, he continued to have significant neurological deficits. One year after the initial presentation, he was undergoing outpatient physical and occupational therapy.

## Conclusions

We presented a case of neurocysticercosis in a patient in the United States without any travel history. Neurocysticercosis should be kept as a differential diagnosis of all seizure cases even in the nonendemic regions without relevant travel history. Multiple neurocysticercosis infestations can present with an acute encephalitic picture, complicating the diagnosis.
